# Ferroptosis-related biomarkers for adamantinomatous craniopharyngioma treatment: conclusions from machine learning techniques

**DOI:** 10.3389/fendo.2024.1362278

**Published:** 2024-11-13

**Authors:** Ying Feng, Zhen Zhang, Jiahao Tang, Yan Chen, Dan Hu, Xinwei Huang, Fangping Li

**Affiliations:** Department of Endocrinology, The Seventh Affiliated Hospital of Sun Yat-sen University, Shenzhen, China

**Keywords:** Adamantinomatous craniopharyngioma, machine learning, ferroptosis, biomarkers, diagnosis

## Abstract

**Introduction:**

Adamantinomatous craniopharyngioma (ACP) is difficult to cure completely and prone to recurrence after surgery. Ferroptosis as an iron-dependent programmed cell death, may be a critical process in ACP. The study aimed to screen diagnostic markers related to ferroptosis in ACP to improve diagnostic accuracy.

**Methods:**

Gene expression profiles of ACP were obtained from the gene expression omnibus (GEO) database. Limma package was used to analyze the differently expressed genes (DEGs). The intersection of DEGs and ferroptosis-related factors was obtained as differently expressed ferroptosis-related genes (DEFRGs). Enrichment analysis was processed, including Gene Ontology (GO), Kyoto Encyclopedia of Genes and Genomes (KEGG), disease ontology (DO), gene set enrichment analysis (GSEA), and Gene Set Variation Analysis (GSVA) analysis. Machine learning algorithms were undertaken for screening diagnostic markers associated with ferroptosis in ACP. The levels of DEFRGs were verified in ACP patients. A nomogram was drawn to predict the relationship between key DEFRG expression and risk of disease. The disease groups were then clustered by consensus clustering analysis.

**Results:**

DEGs were screened between ACP and normal samples. Ferroptosis-related factors were obtained from the FerrDb V2 and GeneCard databases. The correlation between DEFRGs and ferroptosis markers was also confirmed. A total of 6 overlapped DEFRGs were obtained. Based on the results of the nomogram, CASP8, KRT16, KRT19, and TP63 were the protective factors of the risk of disease, while GOT1 and TFAP2C were the risk factors. According to screened DEFRGs, the consensus clustering matrix was differentiated, and the number of clusters was stable. CASP8, KRT16, KRT19, and TP63, were upregulated in ACP patients, while GOT1 was downregulated. CASP8, KRT16, KRT19, TP63, CASP8, and GOT1 affect multiple ferroptosis marker genes. The combination of these genes might be the biomarker for ACP diagnosis via participating ferroptosis process.

**Discussion:**

Ferroptosis-related genes, including CASP8, KRT16, KRT19, TP63, and GOT1 were the potential markers for ACP, which lays the theoretical foundation for ACP diagnosis.

## 1 Introduction

Craniopharyngioma (CP) is a rare epithelial neoplasm, accounting for about 2% to 5% of primary intracranial tumors (1, 2). According to histological typing, there is a higher percentage of adamantinomatous craniopharyngiomas (ACP) compared with papillary craniopharyngioma (PCP) (3). Due to its unique location, complications, such as hypothalamic dysfunction, endocrine deficiencies, and visual impairment result in a low survival rate of CP patients (4). CP has malignant outcomes. Radiotherapy, stereotactic radiosurgery, internal irradiation therapy, and chemotherapy for the treatment of malignant tumors are used as treatment for craniopharyngiomas (5, 6). However, the risk of high recurrence rates and complications is always present (7). More effective treatments need to be researched.

Molecular biology techniques provide more theoretical support for genetic alterations in CPs, facilitate the identification of different biomarkers, and offer new perspectives for target treatment (8). As reported in a previous study, mutations in the Catenin Beta 1 (CTNNB1) cause the deposition of β-chain proteins in the cytoplasm of the nucleus, which activates the Wingless-Type MMTV Integration Site Family (WNT)/β⁃catenin signaling pathway, leading to ACP occurrence (9). β⁃catenin mutations activate the WNT pathway and cause alterations in the MEK/ERK pathway, resulting in proliferation and invasion of ACP cells (10). These ACP-specific genes have expanded therapeutic selectivity and provided the basis for individualized treatment. Further translational research and clinical trials are in progress, and more effective targets are needed to provide a theoretical basis for drug development.

Ferroptosis is an iron-dependent and programmed cell death distinct from apoptosis, necrosis, and autophagy (11, 12). In recent years, it plays an important role in a variety of diseases, including cancer (13), neurodegeneration (14) and ischemic organ damage (15). Although ferroptosis has been studied in various diseases, its mechanism in ACP remains unclear. Traditional methods for marker screening included single-factor statistical analysis, multi-factor statistical analysis, and single machine learning methods (16). Weak screening ability, the complex panel of markers, and poor accuracy of markers limited the screening ability. To screen potential biomarkers with high sensitivity, accuracy, and stability, multiple algorithms of machine learning methods for marker screening were applied (17). Machine learning algorithms have been used to screen precise biomarkers for the treatment of various diseases (18). In this study, we screened differently expressed ferroptosis-related genes (DEFRGs) via machine learning techniques, aiming to provide a ferroptosis-related therapeutic target and further lay the theoretical foundation for the ACP treatment.

## 2 Materials and methods

### 2.1 Data collection

Gene expression profiles, including GSE68015 (19) and GSE94349 (20) were obtained from the gene expression omnibus (GEO) database (https://www.ncbi.nlm.nih.gov/geo/). Therefore, the GSE68015 database contains 15 ACP samples and 16 normal brain tissue samples. The GSE94349 database contained 9 ACP and 17 normal brain tissue samples. Using the “sva” R package, we merged the two datasets. There are 24 ACP and 33 normal brain tissue samples. The platform was Affymetrix HG-U133plus2 chips (Platform GPL570). Through the “sva” R package, the two data sets were merged and normalized. To eliminate the batch effect, we normalized the gene expression matrices of the two datasets and removed the genes that were missing from each other. The ComBat function of the SVA package in R was used to eliminate the batch effect.

### 2.2 Screening of DEFRGs

Limma package of R language was used to screen different expressed genes (DEGs) between 24 ACP and 33 normal brain tissue samples with the threshold of |LogFC| > 2 and P < 0.05. Then, the ggVolcano package and TBtools were used to show the heatmap and volcano plot, respectively. We obtained ferroptosis-related factors from the databases of FerrDb V2 (zhounan. org/ferrdb/current/) and GeneCard [GeneCards - Human Genes | Gene Database | Gene Search (weizmann. ac. il)]. Then, the intersection of DEGs and ferroptosis-related factors was obtained as DEFRGs for further analysis, which was shown by the Venn diagram. Furthermore, protein-protein interaction (PPI) analysis of DEFRGs was conducted based on the STRING website (STRING: functional protein association networks (string db. org) to demonstrate the regulatory relationships.

### 2.3 Enrichment analysis of DEGs

According to the database of DAVID (http://david.ncifcrf.gov/), the candidate DEFRGs were processed for GO annotations. FunRich tool (http://www.funrich.org/) was undertaken for the Kyoto Encyclopedia of Genes and Genomes (KEGG) analysis. Disease Ontology (DO) enrichment was conducted via OmicShare tools (www.omicshare.com) The enrichment list with a P-value < 0.05 was screened with statistical significance. Msigdb database of gene set enrichment analysis (GSEA, gsea-msigdb.org) was used to analyze the enriched KEGG pathways. We used the GSVA package of R software to analyze up- and down-regulated DEGs.

### 2.4 Machine learning techniques for DEFRG screening

Least absolute shrinkage and selection operator (LASSO) and support vector machine recursive feature elimination (SVM-RFE) are two important algorithms of machine learning techniques. Lasso linear regression (21–23) is capable of feature selection and regularization. The basic principle is to add the absolute value of the model weight coefficients to the loss function. The overfitting of the model is prevented and the generalization ability of the model is increased by reducing the weights and lowering the weight values. The SVM-RFE algorithm (24) is a combination of SVM and RFE algorithms. Importance assessment of features was performed by SVM model and unimportant features were eliminated using the iterative process of RFE. The SVM-RFE algorithm was based on the principle of maximum interval of SVM, and scores of each feature are ranked by model training samples. The features with the smallest feature scores were removed using the RFE algorithm in a step-by-step iterative manner. Then, the remaining features were used to train the model again for the next iteration, and the optimal feature combination was selected. In this study, we used these algorithms to screen DEFRGs. e1071 and glmnet packages were used for SVM-RFE and LASSO algorithms, respectively. The screened genes were characterized by higher discriminative power and variable, respectively. The overlapped genes were obtained as key DEFRGs. Correlation analysis between DEFRGs was undertaken, and visualized by ggplot 2.

### 2.5 Patients and tissue collection

The study was approved by the Medical Ethics Committee of The Seventh Affiliated Hospital of Sun Yat-sen University (2019SYSUSH-020). A total of 35 ACP patients were included. All patients were hospitalized and underwent surgery in our hospital from Mar 2018 through May 2022, and they did not receive radiotherapy and chemotherapy treatments. A total of 10 normal brain samples as controls were obtained by autopsies. All patients or their family were informed and consented. ACP and control tissues were obtained and refrigerated at −80°C.

### 2.6 Quantitative polymerase chain reaction (Q-PCR) assay

Purlink RNA Mini assay kit (Thermo Fisher Scientific, Waltham, MA, USA) was used for isolating the total RNA. Then, the obtained RNA was reversed and transcribed into complementary DNA (cDNA). The primers were designed and obtained from Sangon Biotech (Shanghai, China). These are shown as follows:

CASP8, (F) 5′-ATGGCTACGGTGAAGAACTGCG-3′,
(R) 5′-TAGTTCACGCCAGTCAGGATGC-3′;
GOT1, (F) 5′-CTGGGAGTGGGAGCATAT-3′,
(R) 5′-CAAGGGCAAGACGAGAAG-3′;
KRT16, (F) 5′- GAGATCAAAGACTACAGCCC-3′,
(R) 5′-CATTCTCGTACTTGGTCCTG-3′;
KRT19, (F) 3’-GCACTACAGCCACTACTACACGA-5’,
(R) 3’-CTCATGCGCAGAGCCTGTT-5’;
TFAP2C, (F) 5′-ATCGAAAAATGGAGGCCGGT-3′,
(R) 5′-CGGCTTCACAGACATAGGCA-3′;
TP63 (F) 5’-CAATGGCTGGAGACATGAATGGACTCA-3’,
(R) 5’-CTGCCTTCTGTGAGCCAGCTTATCAACC-3’.

The SuperReal PreMix Color kit (Tiangen, Beijing, China) was applied to detect the expressions of the above genes. The 2^-ΔΔCt^ was applied to show the expression levels of key DEFRGs. β-actin was used as a reference.

### 2.7 Enzyme-linked immunosorbent assay (ELISA)

Ferroptosis markers, including SLC40A1, NFE2L2, HSPB1, GPX4, CHAC1, PTGS2, TF, TFRC, and FTH1 were detected using ELISA kits (Multi Sciences Co., Ltd., China) according to the instructions. The steps were as follows. A total of 20 mg of fresh tissue was obtained and used to detect the expression levels of ferroptosis markers, including SLC40A1, NFE2L2, HSPB1, GPX4, CHAC1, PTGS2, TF, TFRC, and FTH1. The homogenizing medium was added and centrifuged at 4°C, 1500×g for 10 min. The middle layer was aspirated for the experiment. Specific anti-mouse antibodies were pre-coated on an enzyme-labeled plate. The wells of the plate were filled with a standard, the sample to be tested, and a biotinylated detection antibody. After incubation, the unbound antibody was removed and horseradish peroxidase-labeled streptavidin (Streptavidin-HRP) was added. TMB was added and the color was developed avoiding light. After color development, samples were detected by Automatic Microplate Reader (MuhiskanMk3, Thermo Labsystems Ltd., USA).

### 2.8 Statistical analysis

R (version 4.1.2) was used for statistical analyses. Receiver operating characteristic (ROC) curves were applied to validate the accuracy of machine learning algorithms. Student *t*-test was used for comparison between the two groups. P < 0.05 was the threshold of significance. All experiments were repeated more than 3 times. According to the multivariate Cox proportional hazards regression model, a nomogram was drawn to predict the relationship between key DEFRG expression and risk of disease. The disease groups were clustered by consensus clustering analysis. The correlation analysis between 6 genes with 9 Ferroptosis related markers, including SLC40A1, NFE2L2, HSPB1, GPX4, CHAC1, PTGS2, TF, TFRC, and FTH1 (25), was calculated using the Pearson correlation method. The statistical software used was Statistical Package for the Social Sciences (SPSS) 20.0.

## 3 Results

### 3.1 Identification of DEFRGs

**Figure 1:**
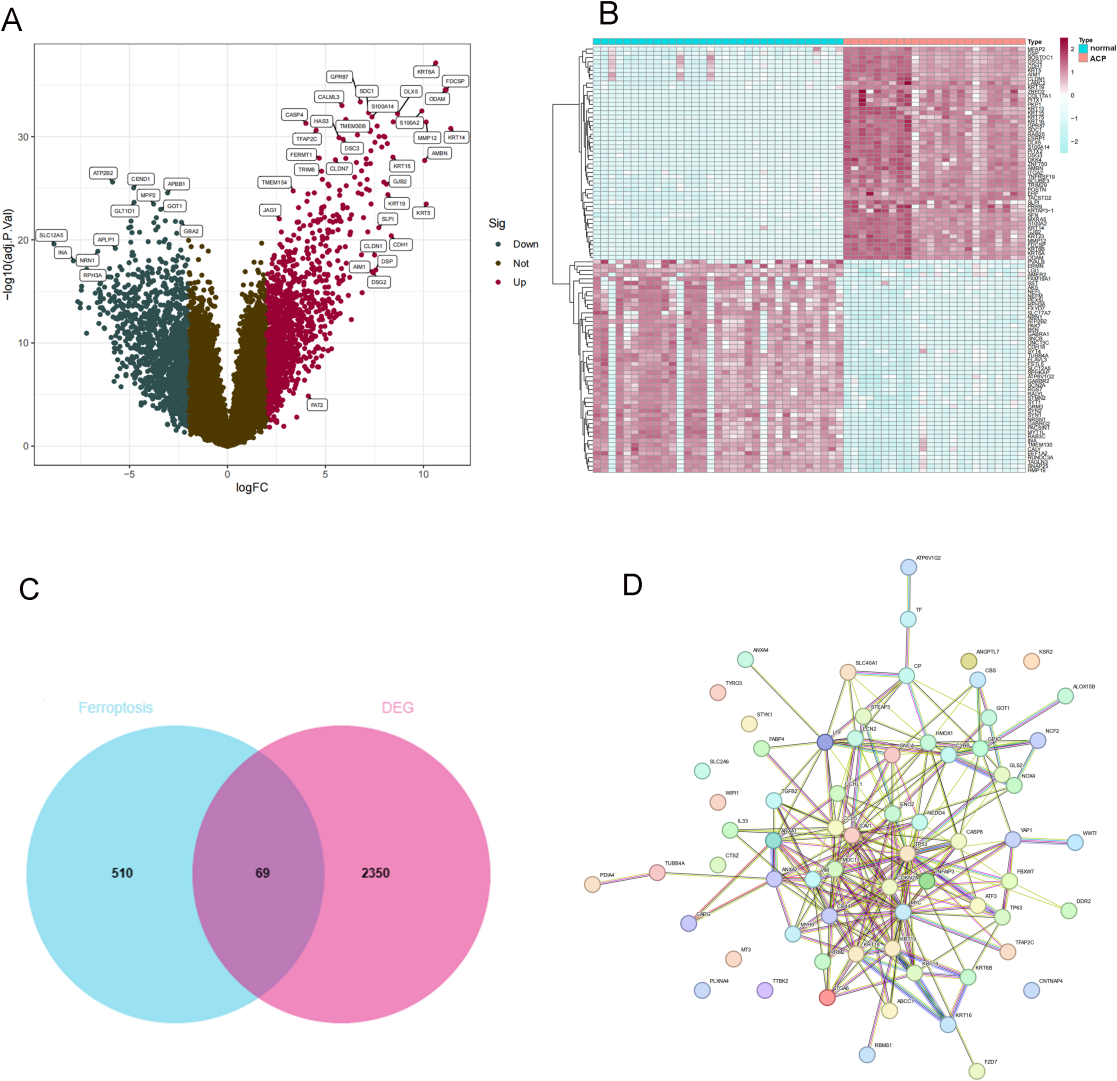
Identification of DEGs between ACP sample and controls. **(A)** Volcano plot analysis. **(B)** The heatmap of DEGs. **(C)** The Venn diagram for screening DEFRGs. **(D)** PPI Networks of DEFRGs. **(C)** Venn diagram for identification of DEFRGs. **(D)** PPI network analysis of DEFRGs.

GSE68015 and GSE94349 were obtained from the GEO database (Home - GEO - NCBI (nih.gov)). GSE68015 contains 15 ACP and 16 normal brain tissue samples. GSE94349 contains 9 ACP and 17 normal brain tissue samples. Using the “sva” R package, we merged the two datasets and normalized them. According to the threshold of | LogFC |>2 and P < 0.05, differential analysis was conducted on 24 ACP and 33 normal brain tissue samples. We obtained 2419 DEGs, including 1181 upregulated DEGs and 1238 downregulated DEGs. The heatmap and volcano plot of these DEGs are shown in **Figures 1A, B**. The intersection of DEGs and ferroptosis-related factors was shown by the Venn diagram. A total of 579 ferroptosis-related factors were obtained from the FerrDb V2 and GeneCard databases (**Figure 1C**). Venn diagram showed that a total of 69 DEFRGs were identified. Then, PPI network analysis was processed to show the regulated relationship of these DEFRGs (**Figure 1D**).

### 3.2 Enrichment analysis of DEGs

**Figure 2:**
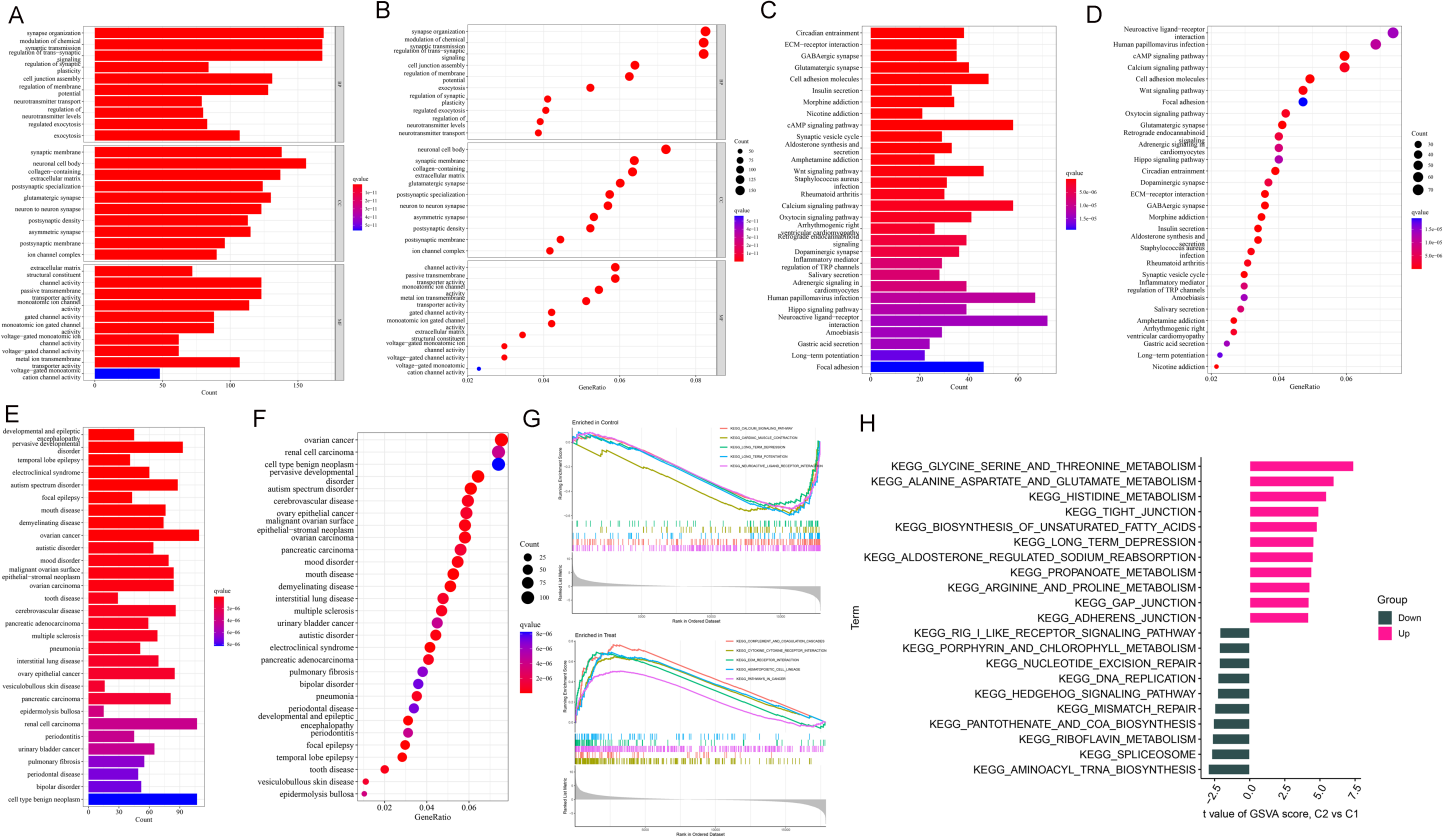
Enrichment analysis of DEGs. **(A, B)** GO analysis. **(C, D)** KEGG analysis. **(E, F)** DO analysis. **(G)** GSEA snapshots of KEGG pathway enrichment analysis. **(H)** GSVA analysis for up- and down-regulated DEGs.

According to the results of GO functional and KEGG pathway enrichment analysis, DEGs were enriched in various functions, such as synapse organization, synaptic membrane, neuronal cell body, and channel activity (**Figures 2A, B**). KEGG pathways enriched by DEGs included circadian entrainment, ECM-receptor interaction, GABAergic synapse, and glutamatergic synapse (**Figures 2C, D**). As shown in **Figures 2E, F**, these DEGs were enriched into many diseases, including pervasive developmental disorder, ovarian cancer, renal cell carcinoma, and cell-type benign neoplasm. Based on the results of the GSEA analysis, critical pathways were obtained. A total of 5 KEGG pathways were enriched, including calcium signaling pathway, cardiac muscle contraction, long-term depression, long-term potentiation, and neuroactive ligand-receptor interaction. Besides, there were 5 KEGG pathways obtained in the disease, including complement and coagulation cascades, cytokine receptor interaction, ECM receptor interaction, hemopoietic cell uncage, and pathways in cancer (**Figure 2G**). Then, GSVA analysis was processed for up- and down-regulated DEFRGs. The up-regulated DEGs were enriched in various pathways, such as glycine serine and threonine metabolism, alanine aspartate and glutamate metabolism, histidine metabolism, and tight junction. While down-regulated DEGs were involved in different KEGG pathways, including aminoacyl trna biosynthesis, spliceosome, and riboflavin metabolism (**Figure 2H**).

### 3.3 Identification of variable DEFRGs with higher discriminative power

**Figure 3:**
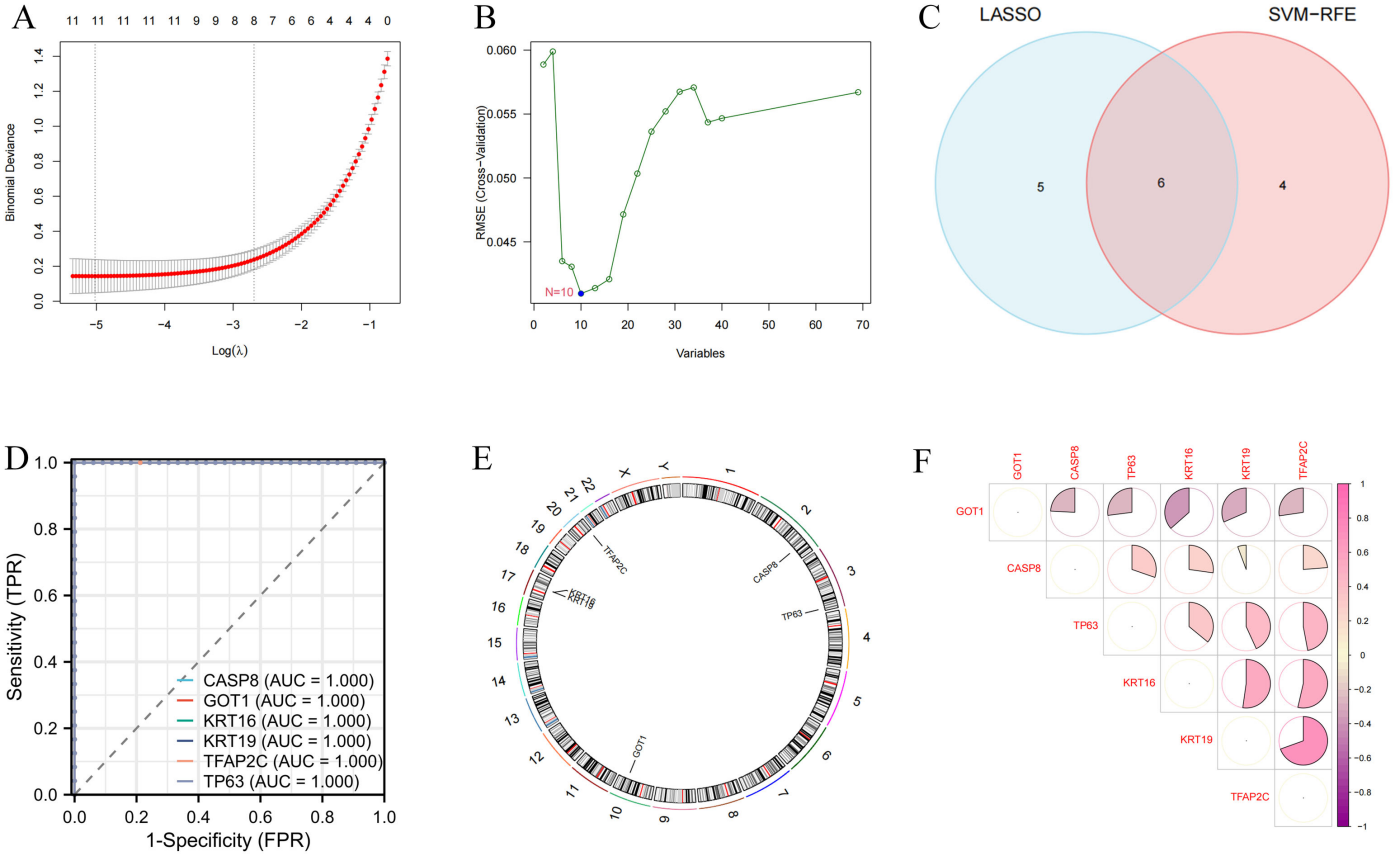
Identification of variable DEFRGs by machine learning techniques. **(A)** Lasso regression analysis. **(B)** SVM analysis. **(C)** Overlapped DEFRGs. **(D)** The ROC curves of the six diagnostic markers. **(E)** Location of diagnostic markers in human chromosomes. **(F)** Correlation of six diagnostic markers. The shades of color had a positive relationship with correlation.

SVM-RFE and LASSO algorithms were used for the identification of variable DEFRGs with higher discriminative power. As the results are shown in **Figures 3A, B**, a total of 11 and 10 features were obtained by SVM-RFE and LASSO algorithms, respectively. There were 6 overlapped DEFRGs were obtained, including CASP8, GOT1, KRT16, KRT19, TFAP2C, and TP63 (**Figure 3C**). The ROC curves of the six diagnostic markers are shown in **Figure 3D**. Their AUC values showed the accuracy of the machine learning algorithm. These Overlapped DEFRGs were located on chromosomes 2, 3, 10, 17 and 20 (**Figure 3E**). The correlation between 6 DEFRGs was calculated. GOT1 was negatively correlated with other DEFRGs, while KRT16, KRT19, and TFAP2C were with positive regulate relationships (**Figure 3F**).

### 3.4 The expression levels of key DEFRGs based on GSE68015 and GSE94349 profiles

**Figure 4:**
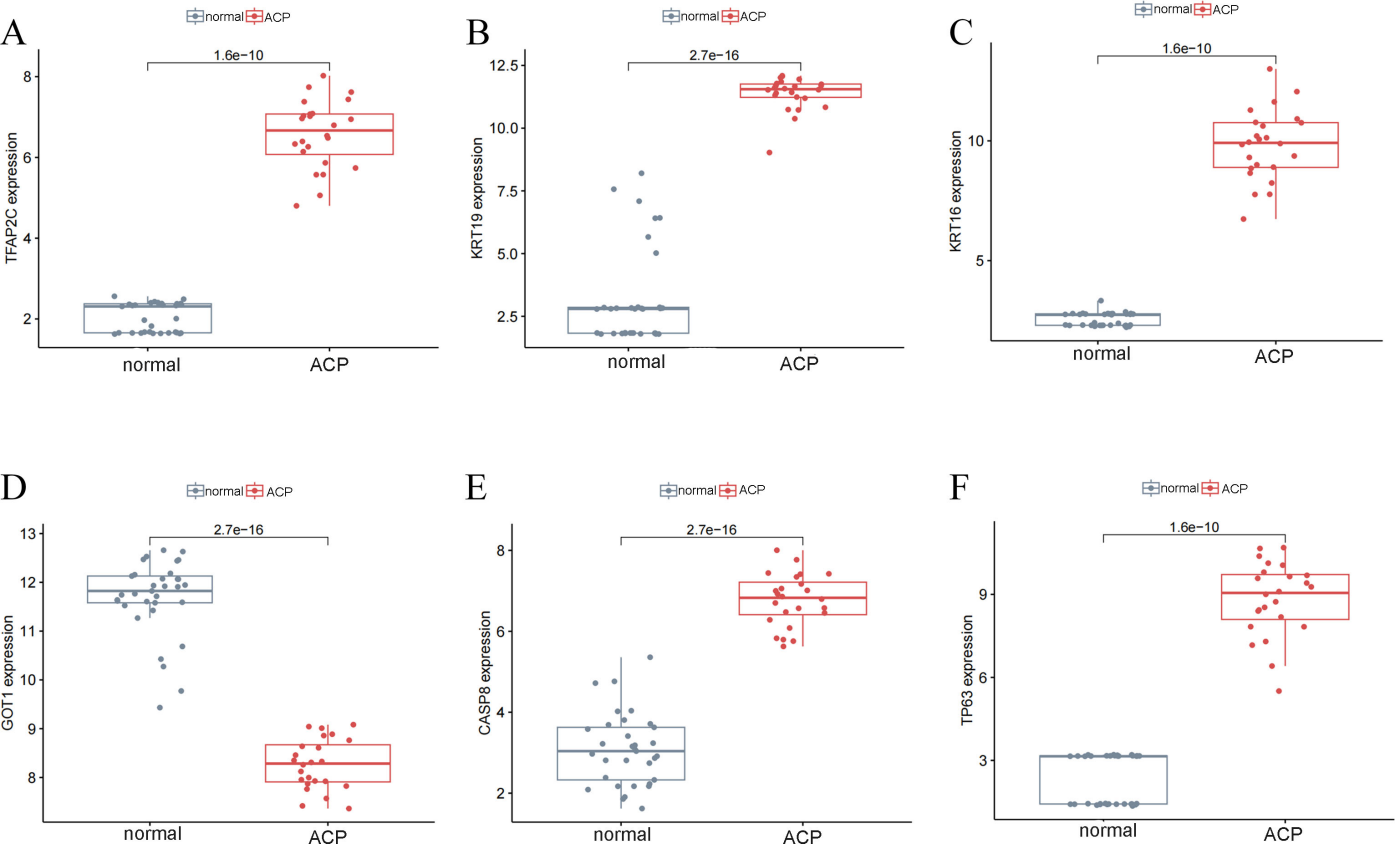
The expression levels of key DERGs in ACP patients based on GSE68015 and GSE94349 profiles. **(A–F)** The levels of CASP8, KRT16, KRT19, TFAP2C, GOT1 and TP63.

These 6 key DEFRGs were differentially expressed in the normal and ACP disease groups based on GSE68015 and GSE94349 profiles. **Figures 4A–F** showed that all five markers, including CASP8, KRT16, KRT19, TFAP2C, and TP63, were upregulated in the disease group, while GOT1 was downregulated in the ACP group.

### 3.5 Prediction of key DEFRGs for ACP

**Figure 5:**
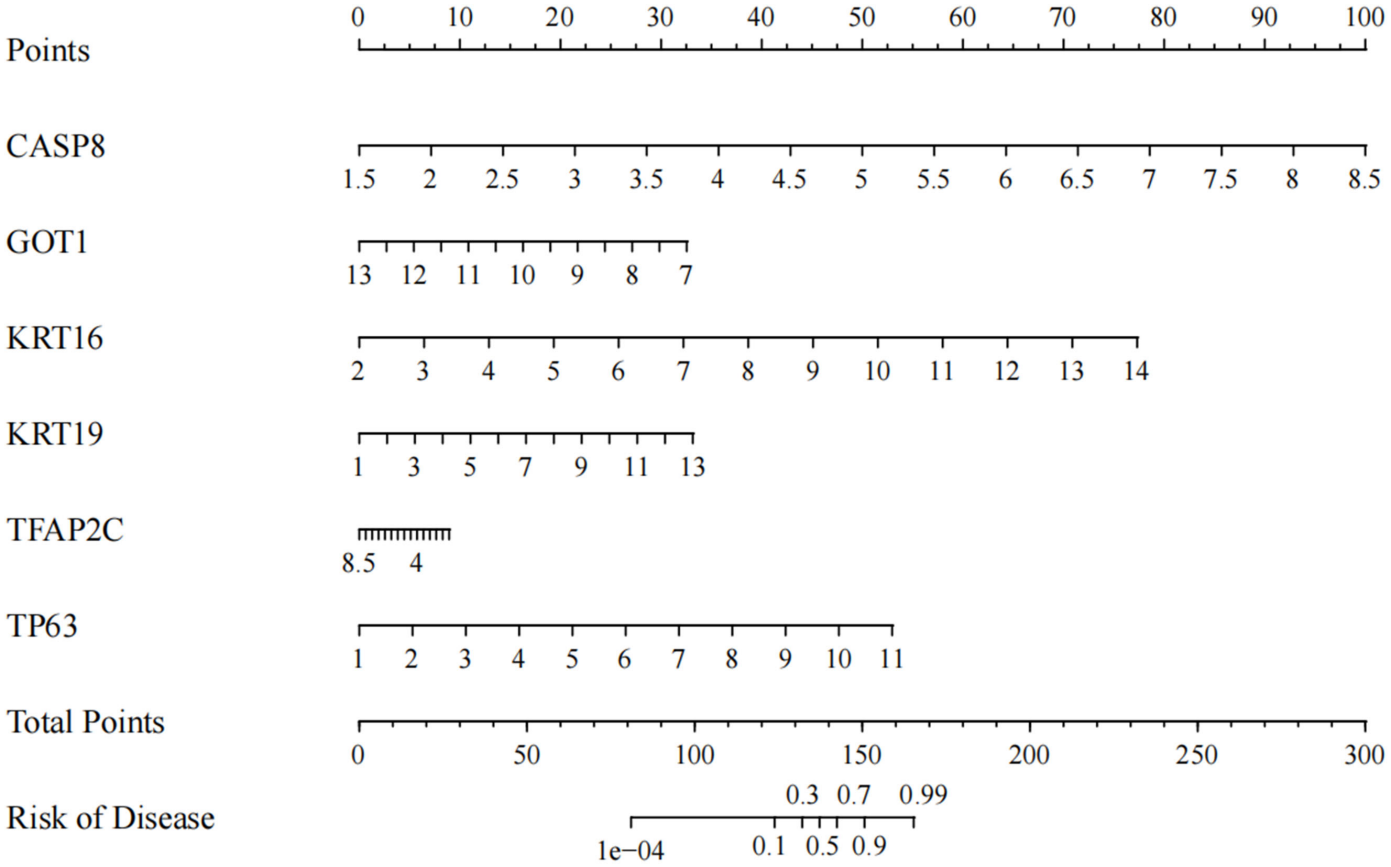
Nomogram to predict CASP8, KRT16, KRT19, TFAP2C, TP63 and GOT1 for ACP patients.

In ACP, total key DEFRGs as diagnostic markers were screened by machine learning. The risk profile of the disease was predicted by the expression levels of these six regulators. Based on the results of the nomogram, CASP8, KRT16, KRT19, and TP63 were the protective factors of the disease, while GOT1 and TFAP2C were the risk factors (**Figure 5**). Except for TFAP2C, the other 5 DEFGRs were composite clinical expression features. CASP8, KRT16, KRT19, TP63, and GOT1 were potential biomarkers for ACP disease.

### 3.6 Consensus clustering analysis

**Figure 6:**
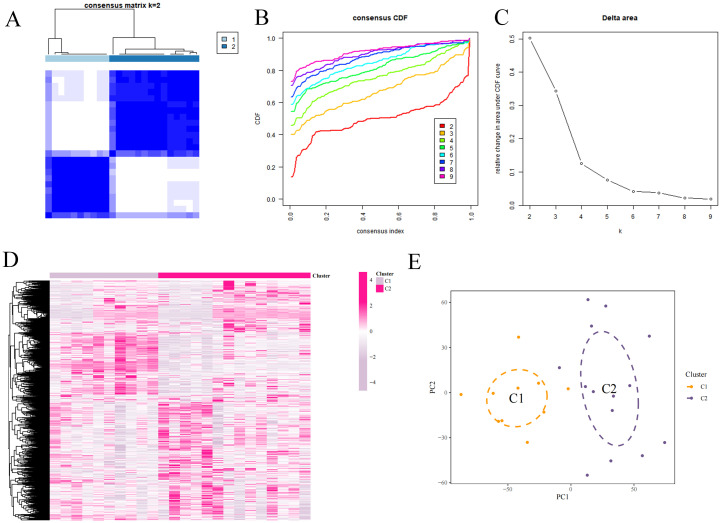
Consensus clustering analysis. **(A)** Consensus matrix. **(B)** Cumulative distribution function of consistency. **(C)** Delta Area Plot. **(D)** Mixture coefficients. **(E)** Cluster analysis.

The samples in the dataset were clustered according to the screened DEFRGs. When the k value was set to 2, the consensus clustering matrix was the most differentiated, the number of clusters was the most stable, and the consistency scores across subtypes were the highest. The samples could be categorized into 2 isoforms and heat mapped, which showed that C2 expressed higher (**Figures 6A–E**).

### 3.7 The expression levels of key DERGs in ACP patients

**Figure 7:**
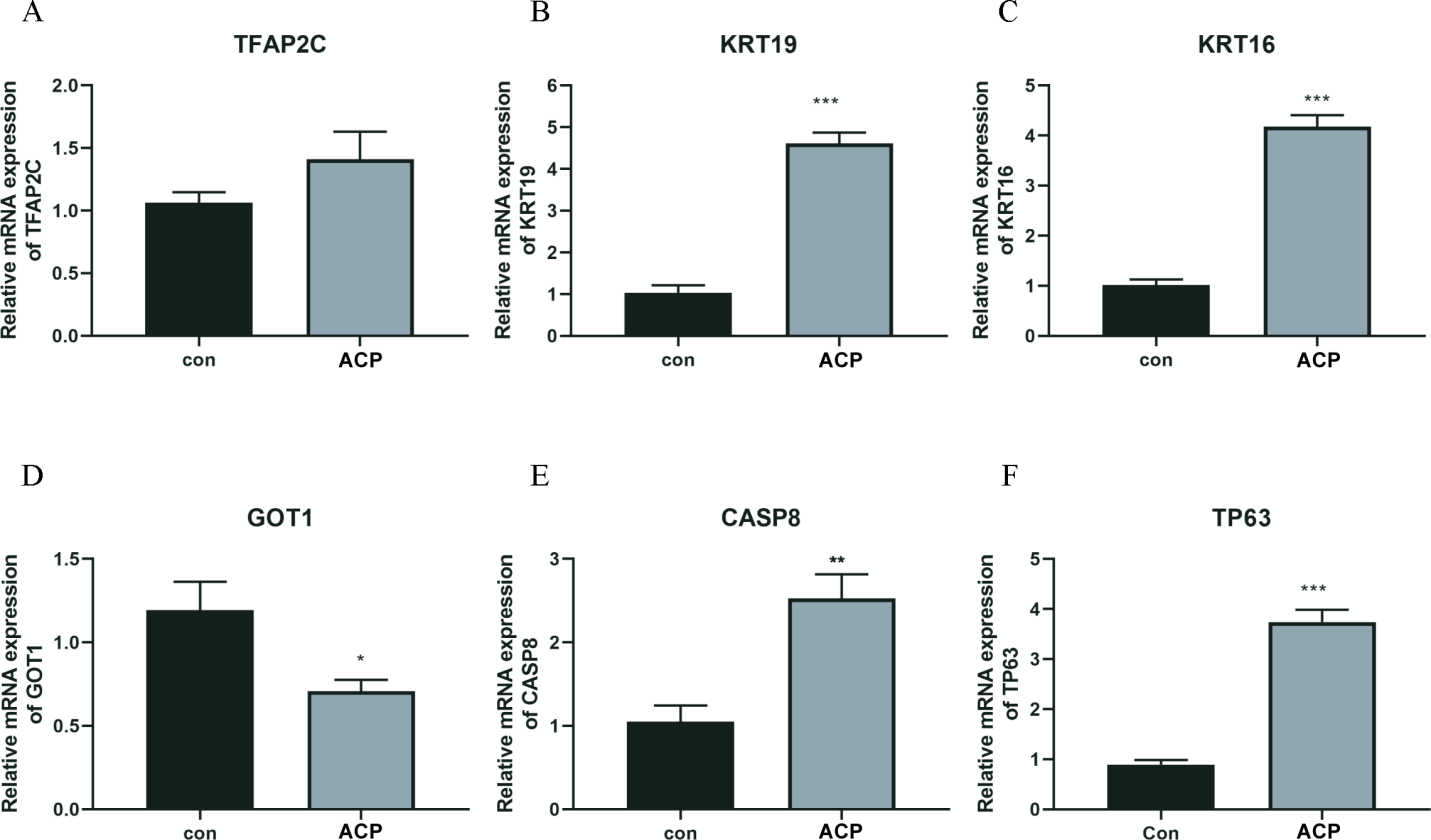
The expression levels of key DERGs in ACP patients **(A–F**). The expression levels of TFAP2C, KRT19, KRT16, GOT1, CASP8, and TP63 in ACP patients detected by q-PCR. *P < 0.05, **P < 0.01, ***P < 0.001.

A total of 5 key DEFRGs were differentially expressed in the normal and ACP disease groups. The results of **Figures 7A–F** showed that CASP8, KRT16, KRT19, and TP63, were upregulated in the disease group, while GOT1 was downregulated in the ACP group. However, TFAP2C was with no significant difference. CASP8, KRT16, KRT19, TP63, and GOT1 might be the biomarker for ACP diagnosis.

### 3.8 Screened key DEGs were significantly related to ferroptosis

**Figure 8:**
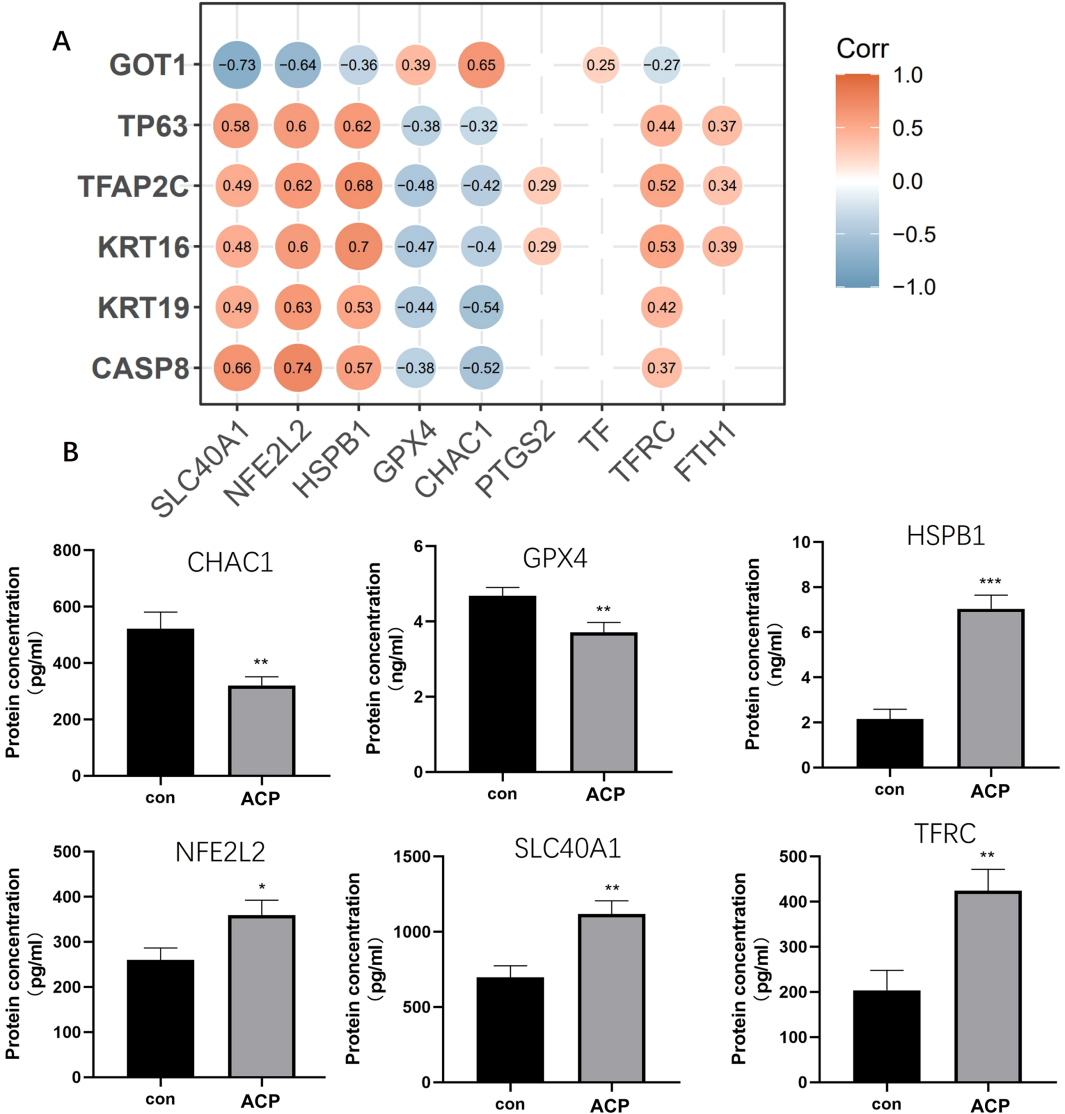
Screened key DEGs were significantly related to ferroptosis. **(A)** The correlation of the six key DEGs with nine ferroptosis marker genes. **(B)** ELISA assay was used for detecting the expression of 6 ferroptosis marker genes. CHAC1 and GPX4 expressed lower in the disease group than control, while HSPB1, NFE2L2, SLC40A1, and HSPB1 were up-regulated in the disease group. *P < 0.05, **P < 0.01, ***P < 0.001.

The correlation of the six key DEGs with nine ferroptosis marker genes is shown in **Figure 8A**. GPX4, HSPB1, NFE2L2, SLC40A1, CHAC1, and HSPB1 were significantly related to key DEGs. Elisa assay was used for detecting the expression levels of 6 ferroptosis marker genes. CHAC1 and GPX4 expressed lower in ACP disease group than control, while HSPB1, NFE2L2, SLC40A1, and HSPB1 were up-regulated in the disease group (**Figure 8B**). A combination of CASP8, KRT16, KRT19, TP63, and CASP8, GOT1 might be the biomarker for ACP diagnosis via participating ferroptosis process.

## 4 Discussion

Conventional treatment leads to poor complications and adverse reactions in craniopharyngioma development (26). Bioinformatics is applied to craniopharyngioma many times to screen for possible biomarkers, such as immune-related genes (27, 28). Ferroptosis, a mechanism closely associated with the development of various cancers (29–31), has not been studied in ACP. In this study, candidate biomarkers of ferroptosis and ACP were selected by functional enrichment analysis. Then, selecting node genes from the constructed PPI network, and screening candidate biomarkers with three machine learning algorithms were processed. The expression levels of candidate genes were compared, and ROC curves and nomograms were constructed to screen biomarkers with higher accuracy.

In this study, we explored potential therapeutic targets and molecular mechanisms related to ferroptosis in ACP. GSE68015 and GSE94349 databases were downloaded to analyze the DEGs between the ACP sample and normal. Then, ferroptosis-related genes and DEGs were overlapped, and 69 DEFRGs were screened. Based on the result of the PPI network, hub nodes were obtained, such as TP-53, CDKN2A, myc, and MJC1. GO and KEGG enrichment analysis showed that these genes enriched in various functions and pathways, such as ion channel activity, cell junction assembly, ECM-receptor interaction, and calcium signaling pathway. Ferroptosis is the result of a dysfunctional balance between intracellular lipid reactive oxygen production and degradation (32). Ferroptosis is induced by a variety of compounds, and the upstream pathway affects the activity of glutathione peroxidase (GPXs) (33). It decreased cellular antioxidant capacity, leading to an increase in lipid peroxidation and an increase in lipid reactive oxygen species, causing the onset of ferroptosis (34). As reported in a previous study, cell detachment from the extracellular matrix (ECM) is a stress response to ferroptosis (35). Ferroptosis-related functions and pathways correspond to ACP development.

Based on the machine learning algorithm and *in vitro* experiment, a total of 6 diagnostic markers, including CASP8, KRT16, KRT19, TP63, and GOT1 were screened. The ROC curves of the six diagnostic markers were calculated, and the AUC value of all biomarkers was 1, indicating the accuracy of machine learning algorithms. CASP8 is a critical enzyme in the apoptotic pathway (36). Polymorphisms in CASP8 have been associated with the risk of developing a variety of diseases, including gastrointestinal, digestive, colorectal, breast, and lung cancers (37, 38). In ACP patients, CASP8 was up-regulated, and the result was consistent with this study. KRT16 and KRT19 belonged to the keratin family. Keratin is an important component of the epithelial cytoskeleton, with the primary function of maintaining the stability of epithelial cells and tissues (39). They are involved in intracellular signaling pathways (40). In this study, KRT family members were involved in the estrogen signaling pathway. Sex hormone signaling inhibited ferroptosis in cancer cells through MBOAT21/2 mediated PL remodeling (41). TP63 has been confirmed to be up-regulated in ACP (42). Cao et al. (43) referred that 89% of ACP patients were with high levels of TP63. Similar results were obtained in this study. GOT1 was down-regulated in ACP patients. GOT1 accelerated ferritinophagy, and mediated SHK-induced ferroptosis (44). In various diseases, GOT1 inhibits cancer development by ferroptosis (44–46). GOT1 participated in ferroptosis and inhibited pancreatic cancer cell death (45). GOT1-related pathway was associated with abnormal ferroptosis in preeclampsia (47). Based on the results of nomogram in this study, CASP8, KRT16, KRT19 and TP63 were the protective factor of the risk of disease, while GOT1 was the risk factors. CASP8, KRT16, KRT19 and TP63 might be potential markers for ACP diagnose and treatment. CASP8, KRT16, KRT19, TP63, CASP8 and GOT1 affect multiple ferroptosis marker genes in this study. Combination of CASP8, KRT16, KRT19, TP63 and CASP8, GOT1 might be the biomarker for ACP diagnosis via participating ferroptosis process.

The ferroptosis and potential biomarkers (CASP8, KRT16, KRT19, and TP63) were critical for ACP diagnosis. Because ACP is a rare disease, there are few available samples and online data. In further study, we will focus on *in vivo* and *in vitro* experiments. By regulating the expression levels of these genes and ferroptosis-related genes, the influence and corresponding mechanism will be researched in ACP cells and tissues. The relationship between ferroptosis and papillary craniopharyngioma will be researched in the future.

In conclusion, 69 DEFRGs were narrowed down to 5 targets with high biomarker potential through multiple rounds of assays and statistical evaluations by machine learning methods. CASP8, KRT16, KRT19, TP63, and GOT1 were the potential markers for ACP treatment. Ferroptosis was confirmed to be a critical biological process in ACP development. The targets predicted by the machine learning approach are used in the medical diagnosis of ACP, which helps to make more accurate predictions and treatments for patients.

## Data Availability

The datasets presented in this study can be found in online repositories. The names of the repository/repositories and accession number(s) can be found in the article/supplementary material.
